# Establishing a Virus-Induced Gene Silencing System in *Lycoris chinensis*

**DOI:** 10.3390/plants12132458

**Published:** 2023-06-27

**Authors:** Guanghao Cheng, Xiaochun Shu, Zhong Wang, Ning Wang, Fengjiao Zhang

**Affiliations:** 1 Institute of Botany, Jiangsu Province and Chinese Academy of Sciences, Nanjing 210014, China; chengghao@yeah.net (G.C.); sxc@cnbg.net (X.S.); wangzhong@cnbg.net (Z.W.); wangning813@njau.edu.cn (N.W.); 2 Jiangsu Key Laboratory for the Research and Utilization of Plant Resources, Nanjing 210014, China; 3 Nanjing Botanical Garden Mem. Sun Yat-Sen, Nanjing 210014, China

**Keywords:** *Lycoris*, VIGS system, leaf tip injection, infiltration efficiency, indicator genes

## Abstract

*Lycoris* is an important plant with both medicinal and ornamental values. However, it does not have an efficient genetic transformation system, which makes it difficult to study gene function of the genus. Virus-induced gene silencing (VIGS) is an effective technique for studying gene functions in plants. In this study, we develop an efficient virus-induced gene-silencing (VIGS) system using the leaf tip needle injection method. The widely used TRV vector is constructed, and the *Cloroplastos Alterados 1* (*CLA1*) and *Phytoene Desaturase* (*PDS*) genes are selected as visual indicators in the VIGS system. As a result, it is observed that leaves infected with TRV-*LcCLA1* and TRV-*LcPDS* both show a yellowing phenotype (loss of green), and the chlorosis range of TRV-*LcCLA1* was larger and deeper than that of TRV-*LcPDS*. qRT-PCR results show that the expression levels of *LcCLA1* and *LcPDS* are significantly reduced, and the silencing efficiency of *LcCLA1* is higher than that of *LcPDS*. These results indicate that the VIGS system of *L. chinensis* was preliminarily established, and *LcCLA1* is more suitable as a gene-silencing indicator. For the monocotyledonous plant leaves with a waxy surface, the leaf tip injection method greatly improves the infiltration efficiency. The newly established VIGS system will contribute to gene functional research in *Lycoris* species.

## 1. Introduction

*Lycoris* belongs to the Amaryllidaceae family and consists of around 20 distinct species worldwide. They are native to Eastern Asia and distributed in moist warm temperature areas, especially in China, Japan and Korea [1]. Most species are valued for their stunningly vibrant and distinct coloration and striking blooms. As an important class of bulbous flowers, they also have low environmental requirements and strong adaptability, making them a good choice for landscaping [2]. In addition, the bulbs contain a large number of alkaloid compounds, which have anti-malarial and anti-tumor effects and are a treatment for senile dementia [3,4], so the plants also have important medicinal value. Due to the great ornamental and medicinal value of *Lycoris*, more and more transcriptomic data have been provided, and functional genes related to excellent breeding traits have been identified [5,6,7,8]. However, due to the lack of efficient genetic transformation, there has been no report on the gene function of *Lycoris* thus far. Moreover, *Lycoris* is a perennial bulbous flower; it takes 3–5 years to grow from a small bulb to a flowering plant [9]. In the study of the functional genes of flowering traits, a long period is always needed to observe the phenotype after conventional genetic transformation. Therefore, it is necessary to establish an efficient, rapid and appropriate transformation system to promote research on the molecular regulation mechanism of this genus.

Virus-induced gene silencing (VIGS) is an excellent alternative to obtaining information about gene function by transiently knocking out the gene of interest [10]. It is a post-transcriptional gene silencing (PTGS)-based technology that utilizes natural defense mechanisms employed by plants to protect against invading viruses [11]. Virus-infected plants induce double-stranded RNA-mediated PTGS, which degrades viral RNA [12]. Recombinant viruses carrying partial sequences of host target genes are used to infect and spread throughout the plant [13]. Viral gene transcripts and plant target genes are recognized and degraded by endogenous PTGS, resulting in reduced target gene expression [14]. Compared with other transgenic technologies, VIGS technology can avoid plant transformation and has the advantages of short cycle, low cost and simple operation. At present, it has been applied to a variety of plants for gene function verification, such as Arabidopsis [15], tomato [11], pepper [16], *Lilium* [17], etc. VIGS has great advantages, especially in perennial woody plants and perennial herbaceous plants. In *Vernicia fordii*, VIGS can shorten the time to phenotype observation and identify phenotypes after loss-of-function of a gene of interest within a single generation [14]. For herbaceous plants, the transformation operation of VIGS is simple and fast and can function in different genetic backgrounds [12].

Many viruses have been used to develop VIGS vectors, such as tobacco mosaic virus (TMV) [18], potato virus X (PVX) [19], tomato golden mosaic virus (TGMV) [20], tobacco rattle virus (TRV) [21] and apple latent spherical virus (ALSV) [22]. Among these viral vectors, TRV has been widely used to construct VIGS vectors for silencing target genes in various bulbous perennial flowers; for example, VIGS experiments using TRV vectors in lily petals showed that anthocyanin accumulation was reduced when *LvMYB5* was silenced [23]. After silencing *NtPDS* with TRV vector in Chinese narcissus, extensive chlorosis of leaves was found [24]. However, it is unclear whether TRV vector-based VIGS can be used to reveal gene function in *Lycoris*, and there is no suitable VIGS system established by now.

*Cloroplastos Alterados 1* (*CLA1*) and *Phytoene Desaturase* (*PDS*) are the most commonly used indicator genes for VIGS system establishment. The *CLA1* gene is involved in chloroplast development and has shown a highly pronounced albino phenotype, so the silenced *CLA1* serves as a useful marker to determine silencing efficiency [25]. *CLA1* has mainly been used in cotton as a positive control for VIGS [26]. When the albino phenotype is observed, it indicates that the gene has been silenced. To determine whether *HyPRP1* is required for cotton resistance to Verticillium wilt, the marker gene *CLA1*-VIGS plants are used to determine the silencing efficiency of *HyPRP1* [27]. In upland cotton, using TRV-*CLA1* as a control, it was found that silencing of *GhCLCg-1* resulted in impaired salt tolerance [28]. It has also been successfully used in other species, such as *Arabidopsis* [14] and *Vernicia fordii* [29]. The gene *PDS* also produces an albino phenotype and is widely used as an indicator gene for the VIGS system [30]. The TRV-*PDS* system has been successfully applied in many ornamental plants. For example, in tree peonies, a typical albino phenotype was found in the newly sprouted top leaves of TRV-*PoPDS*-infected triennial tree peony seedlings using leaf syringe infiltration and seedling vacuum infiltration [31]. This shows that TRV-based VIGS technology can be applied to the high-throughput functional characterization of tree peony genes. In *Lilium* × *formolongi* [17], using the inoculation method of rubbing plus injection, albino was observed in newly developing leaves of TRV-*LhPDS*-infected lily seedlings 56 days after infiltration. In *Solanum pseudocapsicum* L. [32], the TRV-*SpPDS* system was used to infect leaves, and obvious albino was found. In *Catharanthus roseus* [33], the TRV-*CrPDS* system was used to infect roots, stems, leaves, and flowers, and it was found that all tissues showed obvious albino, and the phenotypes of leaves and flowers were more obvious. Nishii et al. used a TRV vector with a wide host range to silence the *SrPDS* gene in *Streptocarpus rexii* through *Agrobacterium* inoculation, and finally obtained silenced plants with albino phenotypes [34]. These examples demonstrate the wide application of the TRV-PDS system. However, the *PDS* gene is not only involved in chlorophyll content but also in carotenoid biosynthesis [35]. For example, in highbush blueberry, which is rich in polyphenols and anthocyanins, a phenotype could occur without chlorophyll, but with red coloration after knockout of the *PDS* gene [36]. It suggests that the chlorophyll-lacking phenotype is varied in different species.

The leaves of *Lycoris* plants are linear and have the characteristics of longitudinal and orderly growth of vascular tissue. The surface of the leaves is covered with a thin waxy layer, and thus it is not easy to infiltrate the bacterial solution with the traditional infiltration method. In this study, we developed a leaf tip injection method, constructed TRV vectors using *LcCLA1* and *LcPDS* as reporter genes, and tested the feasibility of the TRV-VIGS system in spring-leafed *L. chinensis*, in which the young leaves emerge from the bulb in early spring when they are suitable for injection. Two weeks after infection, the leaves of *L. chinensis* injected with *LcCLA1* and *LcPDS* showed chlorosis, but the phenotype of leaves injected with *LcPDS* was not as obvious as that injected with *LcCLA1*. The expression level of *LcCLA1* in the chlorotic leaves was significantly lower than that in non-injected leaves. In terms of gene expression, the expression levels of *LcCLA1* and *LcPDS* in chlorotic leaves were significantly lower than those in uninfected leaves, while the gene expression of *LcCLA1* was lower, which indicated that *LcCLA1* was more suitable as an indicator gene in gene function studies. Therefore, TRV-*LcCLA1* can silence the genes in *L. chinensis* more effectively than the TRV-*LcPDS* VIGS system, which lays a good foundation for the gene function verification of *L. chinensis* in the future.

## 2. Results

### 2.1. Comparison of Agrobacterium Infection Efficiency of Different Infiltration Methods

**Figure 1:**
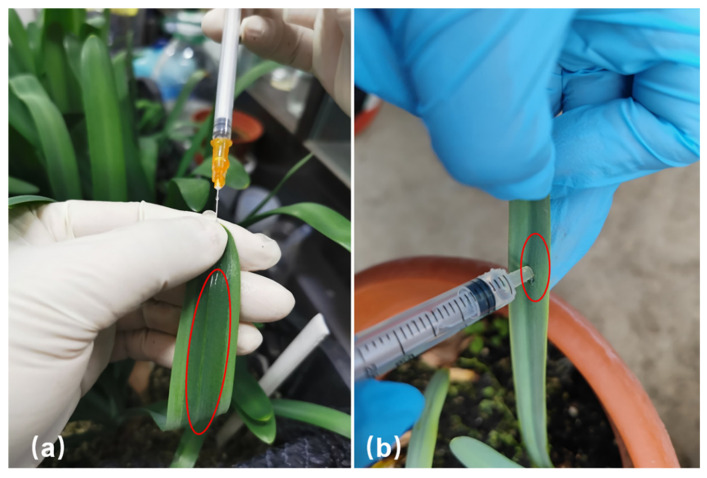
Comparison of tip needle injection method and traditional injection infiltration method. (**a**) Tip needle injection method to infect leaves of *L. chinensis*. (**b**) Traditional infiltration method to infect leaves of *L. chinensis*. The red oval area shows the infection solution that was successfully injected into the leaf.

In this study, we used the tip needle injection method, which requires 1–2 mL bacterial solution and takes 15–20 s to infiltrate a whole leaf (Figure 1a), compared with the conventional leaf infiltration method, which commonly requires at least 5 mL of bacterial solution and takes 1–2 min to infiltrate a leaf (Figure 1b). The reason for such a large difference is the waxy layer on the surface of the leaves of the *Lycoris*. It is difficult to inject *Agrobacterium* solution into the entire leaf when infiltrating. To infiltrate the whole leaf, multiple wounds need to be created. In addition, the infiltration process is very easy to cause the loss of *Agrobacterium* solution, and more wounds are not conducive to normal plant growth and development, thus affecting the experimental results. The tip needle injection method is an easier way to infiltrate the whole leaf, as well as being more efficient, simple to operate, and able to save more solution.

### 2.2. Silencing Efficiency of LcCLA1 and LcPDS in L. chinensis after Infection

**Figure 2:**
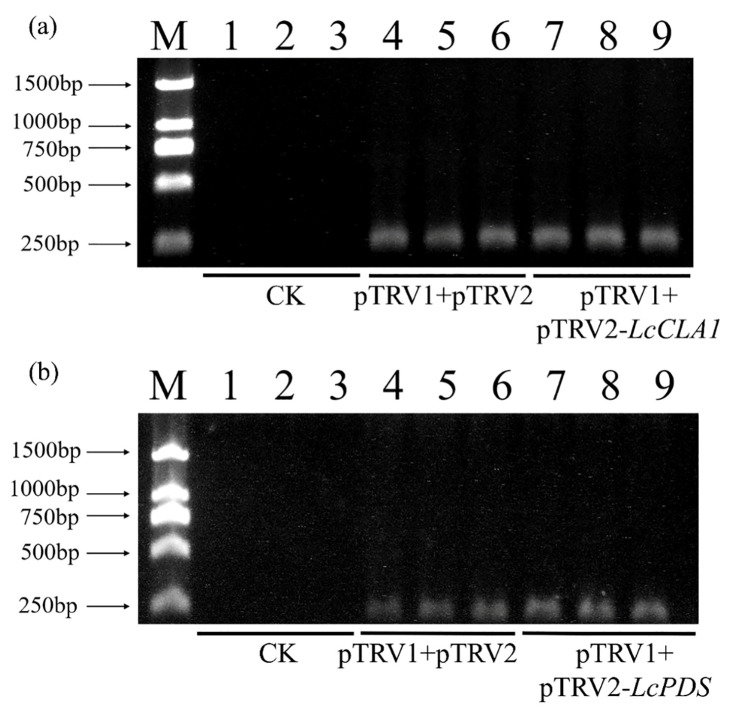
Testing of *LcCLA1* and *LcPDS* injection efficiency. M: marker; 1–3: leaves without injection; 4–6: leaves injected with pTRV1 and pTRV2; 7–9 (**a**): leaves injected with pTRV1 and pTRV2-*LcCLA1*: 7–9 (**b**): leaves injected with pTRV1 and pTRV2-*LcPDS*.

Two weeks after injection, an obvious yellow leaf phenotype was observed in the leaves of *L. chinensis* after injection of pTRV2-*LcCLA1*. The difference is that the leaves injected with pTRV2-*LcPDS* infection solution had less yellow leaf phenotype. Injection efficiency was detected using PCR, and leaves not injected with *Agrobacterium* (CK), pTRV1+pTRV2 (Mock), pTRV1+pTRV2-*LcCLA1* and pTRV1+pTRV2-*LcPDS* were selected for PCR detection. The results showed that no band was seen in CK, while pTRV1+pTRV2 and pTRV1+pTRV2-*LcCLA1* had a clear band at 260 bp (Figure 2a). Similarly, for the injection of *LcPDS*, no band was observed in the CK region, but about 240 bp bands were observed in pTRV1+pTRV2 and pTRV1+pTRV2-*LcPDS* (Figure 2b). This indicates that the tip needle injection method was successful in achieving infection of the leaves of *L. chinensis*.

**Figure 3:**
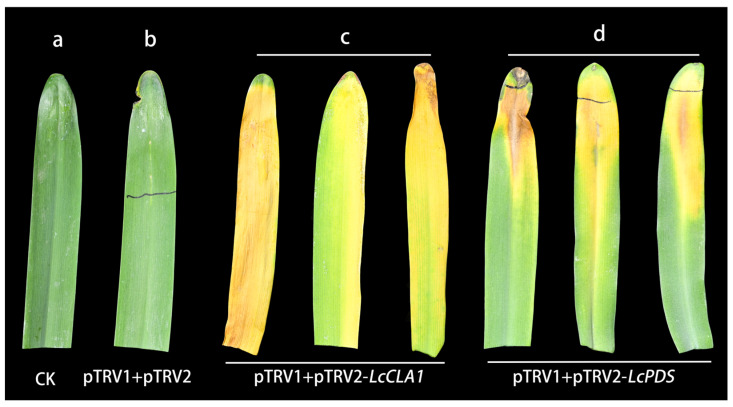
Silencing of *LcCLA1* and *LcPDS* in *L. chinensis* using TRV VIGS vector. (**a**) Leaf of *L. chinensis* without *Agrobacterium* injected as a control (CK). (**b**) Leaf of *L. chinensis* injected with pTRV1 and pTRV2. (**c**) Leaves of *L. chinensis* injected with pTRV1 and pTRV2-*LcCLA1* showed etiolation phenotype after two weeks. (**d**) Leaves of *L. chinensis* injected with pTRV1 and pTRV2-*LcPDS* showed less etiolation phenotype after two weeks.

From the perspective of leaf phenotype, plants inoculated with pTRV1 and pTRV2 showed no significant difference in leaf morphology compared to CK (Figure 3a,b). In comparison with CK, leaves injected with pTRV1+pTRV2-*LcCLA1* were found to show a distinct etiolation phenotype (Figure 3c). However, leaves injected with pTRV1+pTRV2-*LcPDS* showed a weaker etiolation phenotype, which was less pronounced than that of leaves injected with pTRV1+pTRV2-*LcCLA1*. (Figure 3d). Phenotypic differences suggest that the expression of the gene *LcCLA1* may be repressed in leaves infiltrated by pTRV2-*LcCLA1*. The expression level of *LcPDS* might not be completely suppressed because the leaves showed incomplete chlorotic phenotype. The reason for this discrepancy is unclear, but it is speculated that *LcPDS* may have a lower density of *Agrobacterium* and less function in leaves.

### 2.3. qRT-PCR Analysis of LcCLA1 and LcPDS Silencing Level

**Figure 4:**
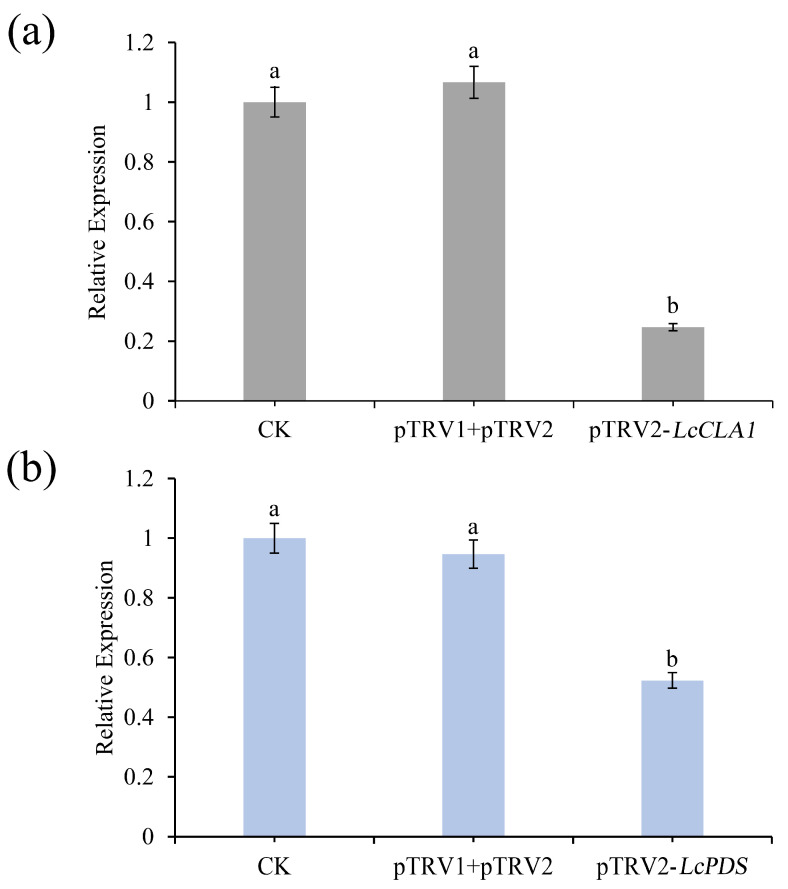
Relative expression levels of *LcCLA1* and *LcPDS* in not injected (CK), TRV vector-injected (pTRV1+pTRV2), pTRV2-*LcCLA1*- and pTRV2-*LcPDS*-injected leaves of *L. chinensis*. (**a**) Relative expression level of *LcCLA1*. (**b**) Relative expression level of *LcPDS*. Error bars represent standard errors, and different lowercase letters indicate significant differences at *p* ≤ 0.05.

After detection of viral infection in *L. chinensis* leaves, we next analyzed gene expression after infection using qRT-PCR. The gene expression of *LcCLA1* and *LcPDS* in untreated wild-type, empty-vector and virus-infected plants was compared to assess gene-silencing efficiency. For both genes, gene expression was significantly reduced in infected *Lycoris* leaves compared to wild-type and empty TRV vector plants (Figure 4). The efficiency of gene silencing was analyzed by monitoring the expression levels of *LcCLA1* in plants showing leaf bleaching phenotype. According to the results, it was found that the expression level of *LcCLA1* in pTRV1- and pTRV2-injected leaves was not significantly changed compared with CK. However, the expression level of *LcCLA1* in leaves injected with pTRV2-*LcCLA1* was about 75% lower than that in CK (Figure 4a). The etiolation phenotype of the leaves was consistent with the expression level of *LcCLA1* after silencing. The expression level of *LcPDS* was also analyzed in leaves injected with pTRV2-*LcPDS* but with less etiolation phenotype. From the results, it was found that the expression level of *LcPDS* in CK leaves was approximately the same as that in leaves injected with pTRV1 and pTRV2. However, the expression level of *LcPDS* in leaves injected with pTRV2-*LcPDS* was only about 50% of that in CK (Figure 4b). Compared with the silencing efficiency of *LcCLA1*, the silencing efficiency of *LcPDS* is much lower.

## 3. Discussion

In this study, we demonstrated that the endogenous genes in *L. chinensis* can be effectively down-regulated using the TRV-VIGS system. Since the current genetic transformation system of *Lycoris* is very immature, it is difficult to rapidly verify the gene function of this genus. Breaking through this barrier would not only take a lot of time and effort but would also need to overcome a lot of technical challenges. Therefore, effective and low-cost technologies need to be developed to temporarily replace genetic transformation systems. We developed the TRV-VIGS system to contribute to the gene regulation research of *Lycoris*, especially in its flowering traits.

*Agrobacterium* infection is the most effective method of virus-based vector infestation of plants [37]. The silencing efficiency varies greatly with different injection methods for the same plant [32]. The silencing efficiency is related to the fluid volume of *Agrobacterium* entering the plant. Since the leaf surface of *Lycoris* has a waxy layer, it is difficult to get the *Agrobacterium* solution into the leaf using the normal method of injection infiltration. We inserted the solution by injection with the needle of a syringe from the tip of the leaf to fully infiltrate the leaf, thus greatly improving the injection efficiency. To visualize whether genes were being knocked down, we used two different reporter genes, *CLA1* and *PDS*, which are commonly used in VIGS and which produce a visible phenotype after successful silencing.

The *CLA1* gene is involved in chloroplast development and is a potent marker in the TRV-VIGS system [38]. The *CLA1* gene was selected as a reporter, and it was tested out to determine if the TRV-VIGS system can be well applied to the study of gene function in *Solanum melongena* [39]. In *Arabidopsis*, the bleaching phenotype after silencing *CLA1* was used as a visible indicator of the silencing efficiency of the TRV-VIGS system [40]. Up to now, *CLA1* has been used as a reporter gene for gene silencing many times in cotton studies. For example, when weather cold-induced changes in non-coding gene expression had a significant effect on the cold tolerance of cotton seedlings, TRV2-*CLA1* was used as a positive control to determine that silencing of lincRNA *XH123* resulted in increased sensitivity to cold injury [41]. When studying the function of the *CLE* gene family in cotton, *CLA* was again used as a positive control, and it was found that the silencing of *GhCLE5* in cotton resulted in dwarf seedlings [42]. Inhibition of *GauGRAS1* by VIGS resulted in glandless stems and petioles of *Gossypium australe* compared with positive control *TRV-CLA* and negative control TRV empty vector [43]. We isolated a homologue of *CLA1* from *L. chinensis* leaves, named *LcCLA1*. Plants were infiltrated with *Agrobacterium tumefaciens* expressing partial sequences of *LcCLA1*, and etiolation phenotype was observed on the injected leaves approximately two weeks after infiltration. qRT-PCR analysis revealed low levels of *LcCLA1* transcript in *L. chinensis* leaves inoculated with the TRV-VIGS system, which confirmed the silencing of this gene. This suggests that the expression of *LcCLA1* is significantly down-regulated in *L. chinensis* through the TRV-VIGS system, resulting in etiolation phenotype of the leaves.

The gene *PDS* encodes an enzyme in carotenoid biosynthesis whose silencing leads to the albino phenotype of plant tissues [18], so it is often used as a marker in VIGS experiments. Up to now, VIGS has been reported to silence *PDS* in many plants, including *Solanum melongena* [39], *Capsicum annuum* [16], tomato [11], *Nicotiana benthamiana* [44], *Sorghum bicolor* [45], soybean [46], etc. In this study, the endogenous *PDS* gene also was selected as the reporter gene in *L. chinensis*. Injected leaves were significantly altered compared to controls, exhibiting a pronounced chlorotic phenotype, especially in the upper and middle sections of leaves. It indicated that the *PDS* gene could be selected as the indicator gene for VIGS system in *L. chinensis*. However, the chlorotic phenotype of *LcCLA1* was more obvious than that of *LcPDS*. qRT-PCR validation also showed that the expression of *LcCLA1* was lower than that of *LcPDS*. The reason may be that the *PDS* gene is not only involved in chlorophyll content but also carotenoid biosynthesis [18,35]. For example, in highbush blueberry that knock out the *PDS* gene, the leaves exhibited a phenotype without chlorophyll, but with red coloration [36]. Thus, our study suggested that the *PDS* gene may also be involved in carotenoid biosynthesis in *Lycoris*, leading to less albino phenotype in leaves.

There are many factors that affect silencing efficiency, such as the growth temperature after inoculation, the growth stage of the inoculated plants, the type of *Agrobacterium* strain, the inoculation method and the inoculum concentration. Different plants require different temperatures to produce a good silencing phenotype after VIGS. In the tomato, the optimal silencing phenotype was obtained when the growth temperature was 22 °C after inoculation [47]. In *S. pseudocapsicum*, silencing efficiency decreased when inoculated seedlings were grown at 18 °C or 30 °C [32]. We need to improve the growth temperature of the VIGS system between 20 °C and 25 °C. The growth stage of the target plant affects silencing efficiency. Gerbera seedlings at the early stages of vegetative development were much more sensitive to TRV VIGS than those at the middle and late stages [48]. We can try to inoculate between one and two weeks after leaf emergence. The optimal *Agrobacterium* strain for VIGS varies from plant to plant and has a strong impact on gene silencing efficiency. Studies have shown that both *Agrobacterium* strains GV2260 and GV3101 can be used in *N. benthamiana*, with GV2260 working best [18]. LBA4404 and GV2260 can be used on the tomato, but the silencing efficiency is very low, and GV3101 has the best silencing effect [49]. We will select LBA4404 and GV2260 to compare with GV3101 and select the strain with the best silencing efficiency. Due to the different inoculation methods and characteristics of different infected plants, the concentration of the infection solution strongly affects the gene silencing efficiency of VIGS experiments. In the TRV-VIGS experiment of *Arabidopsis*, the most suitable concentration of *Agrobacterium* infection solution was OD600 = 1.5, and the silencing efficiency was almost 100% [15]. In addition, naked cotton seeds soaked in *Agrobacterium* inoculum with OD600 of 1.5 for 90 min showed the best silencing efficiency [50]. For vacuum infiltration, some studies have found that the optimal conditions for VIGS are vacuum treatment with *Agrobacterium* liquid with OD600 of 0.3 for 30–60 s, and co-cultivation with the same concentration of *Agrobacterium* for 15 h [51]. The above experiments show that different species require different concentrations of *Agrobacterium* inoculation; different inoculation methods also require different concentrations of *Agrobacterium*. In this study, the silencing of *LcCLA1* and *LcPDS* were successfully achieved by *Agrobacterium* infection at OD600 = 2.0 and cultured at 18–26 °C for 2 weeks. In addition, *L. chinensis* has an obvious chlorosis phenotype. Therefore, this can be used as the recommended concentration for the VIGS system of *Lycoris* plants.

## 4. Materials and Methods

### 4.1. Plant Materials and Infection Methods

*L. chinensis* bulbs were planted in the National Germplasm Bank of *Lycoris*, located in the Institute of Botany, Jiangsu Province and Chinese Academy of Sciences (Nanjing Botanical Garden Mem. Sun Yat-Sen), Nanjing, China. The plants grow in conditions of 12 h/12 h day/night periods and 26 °C/18 °C day/night temperatures. The experiment was performed in March when the leaves were lush. The leaf tip injection method (Figure 1a) was used for bacterial infection, which makes it easier to infiltrate the whole leaf, showing more efficiency than the conventional leaf infiltration method (Figure 1b). After the infection was completed, the plant material was cultured in the dark at 18 °C for 2 d and then grown in a normal environment. After tip injection, when the leaves presented the chlorotic phenotype, the injected leaves and normal growing leaves were collected, labeled as treatment group (T) and control group (CK). All samples were immediately frozen in liquid nitrogen after collection and stored at −80 °C until RNA extraction. The collected leaves were used to analyze the expression of the *LcCLA1* and *LcPDS* genes.

### 4.2. Cloning of the LcCLA1 and LcPDS Genes and VIGS Vector Construction

The gene sequences of *LcCLA1* and *LcPDS* were obtained from transcriptome data of *L. chinensis* [6]. Raw reads were deposited in the NCBI database (https://www.ncbi.nlm.nih.gov/, accessed on 8 June 2022) under BioProject number PRJNA847051. The fresh leaves of *L. chinensis* were collected and put into liquid nitrogen for RNA isolation. Total RNA was extracted using the Plant RNA Extraction Kit (Huayueyang, Beijing, China) according to the manufacturer’s instructions. The RNA concentration and purity were detected using the OneDrop OD1000 system (Wuyi Technology, Nanjing, China) and gel electrophoresis. The cDNA was synthesized using the PrimeScriptTM RT kit with the gDNA Eraser kit (TaKaRa, Dalian, China) according to the manufacturer’s instructions. Primers of *LcCLA1* and *LcPDS* genes (Table 1) were designed using Primier 5 software. The fragments of *LcCLA1* and *LcPDS* were amplified using the designed primers *LcCLA1*-F/R and *LcPDS*-F/R, respectively. The Xba I restriction site was added to the 5′ end of the upstream primer, and the Kpn I restriction site was added to the 5′ end of the downstream primer. The PCR reaction was performed in a 50 μL volume containing 4 μL cDNA, 1 μL dNTP Mix (10 mM), 25 μL 2 × Phanta Max Buffer, 2 μL each of the forward and reverse primers (10 μM), 1 μL Phanta Max Super-Fidelity DNA Polymerase and 15 μL ddH2O. The PCR reaction was performed in the following conditions: denaturation at 95 °C for 3 min, followed by 35 cycles of 95 °C for 15 s, 56 °C for 15 s, 72 °C for 1 min, and finally extension at 72 °C for 5 min.

**Figure 5:**
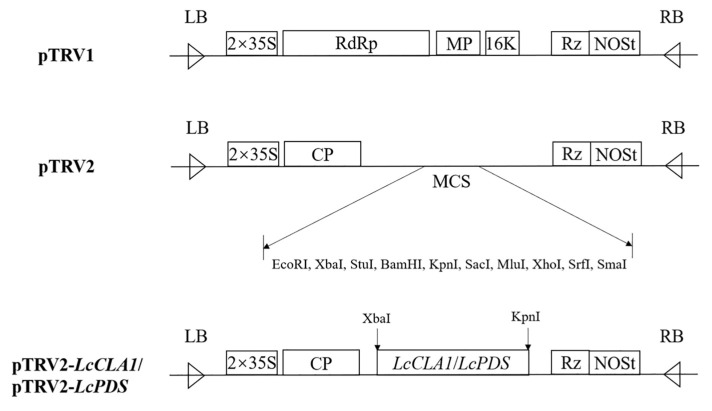
TRV-based VIGS vectors and construction. LB and RB: left and right borders of T-DNA; 2 × 35 S: the duplicated CaMV 35 S promoter; RdRp: RNA-dependent RNA polymerase; MP: movement protein; CP: coat protein; 16 K: 16 kDa cysteine-rich protein; MCS: multiple cloning sites; Rz: self-cleaving ribozyme; NOSt: nopaline synthase terminator.

The TRV-VIGS vectors pTRV1 and pTRV2 were used in this study as reported in previous studies [52]. AxyPrep DNA Gel Extraction Kit (Axygen, Nanjing, China) was used to purify PCR products. After purification, *LcCLA1* and *LcPDS* fragments were assembled into the pTRV2 vector using ClonExpress II one-step cloning kit (Vazyme, Nanjing, China). The specific fragments were respectively attached to pTRV2, and then the silent vectors pTRV2-*LcCLA1* and pTRV2-*LcPDS* were obtained (Figure 5).

### 4.3. Preparation of Agroinfiltration Infection Solution

The plasmids of TRV2-*LcCLA1*, TRV2-*LcPDS*, TRV2 and TRV1 were transformed into *Agrobacterium tumefaciens* strain GV3101 using the freeze-thaw method [53], separately. The transformed plasmids of TRV1, TRV2, TRV2-*LcCLA1* and TRV2-*LcPDS* were cultured in LB solid medium (with 50 μg/mL kanamycin; 25 μg/mL rifampicin). Freshly cultured *A. tumefaciens* GV3101 colonies containing pTRV1, pTRV2, pTRV2-*LcCLA1* and pTRV2-*LcPDS* were selected and added to 3 mL YEB liquid medium (with 50 μg/mL kanamycin; 25 μg/mL rifampicin). Then, they were cultured in a shaker at 28 °C and 170 rpm/min for 16 h. Add 1 mL of the cultured solution to 100 mL of YEB liquid medium (with 50 μg/mL kanamycin; 25 μg/mL rifampicin), and incubate at 28 °C and 170 rpm/min for 20–24 h. When the OD600 of the detected solution is about 1.0, take 40 mL and centrifuge at 4000 rpm for 10 min to collect the cells, and add an appropriate volume of infection buffer to resuspend until the final concentration of OD600 is 2.0. The configuration of the infection buffer was 100 mL of permeate containing 1 mL of MgCl_2_ (1 mol/L), 1 mL of MES (1 mol/L), and 200 μL of acetosyringone (1 mol/L), and the pH was adjusted to 5.6. Make up to volume with sterilized distilled water, which needs to be prepared and used immediately. Two kinds of infection solutions were prepared by mixing pTRV1 with pTRV2 and pTRV2-*LcCLA1* resuspension at a volume ratio of 1:1 [54], respectively. Another infection solution also mixed pTRV1 and pTRV2-*LcPDS* at the same ratio. The mixed infecting solution was placed in a 25 °C shaker at 100 rpm/min and cultured in the dark for 4 h to infect the leaves.

### 4.4. Expression Analysis of LcCLA1 and LcPDS Using qRT-PCR

Leaf chlorosis was observed after 2 weeks, and the leaves of *L. chinensis* that were not injected with infection solution, injected with pTRV1+pTRV2 and injected with pTRV1+pTRV2-*LcCLA1*, pTRV1+pTRV2-*LcPDS* were collected. The collected leaves were immediately frozen in liquid nitrogen and stored at −80 °C for qRT-PCR experiments. The *EXP1* gene was used as an endogenous control to normalize the results. To determine the relative levels of endogenous *LcCLA1* and *LcPDS* transcripts in infected leaves, qRT-PCR was performed using the primer pair EXP1-RT-F, EXP1-RT-R, *LcCLA1*-RT-F, *LcCLA1*-RT-R and *LcPDS*-RT-F, *LcPDS*-RT-R (Table 1). The steps of extracting total RNA, detecting RNA concentration, purity, integrity and reverse transcription are the same as in Section 4.2. qRT-PCR was performed using a StepOnePlus real-time PCR system (Applied Biosystems, Beijing, China) and a 20 µL reaction mixture. Each 20 µL reaction mixture contained 10 µL SYBR Premix Ex TaqTMII (TaKaRa, Dalian, China), 5 µL diluted cDNA, 0.8 µL forward and reverse primer (10 μM), 0.4 µL ROX Reference Dye and 3 µL ddH_2_O. The experiment was repeated three times, and the data were analyzed using the 2^−ΔΔCt^ method to calculate relative gene expression levels [55].

## 5. Conclusions

For species where plant transformation, regeneration and genetic backgrounds are challenging, VIGS provides a powerful tool for transient gene function studies. We demonstrated that the TRV-based VIGS system can effectively silence genes in *L. chinensis* and established a TRV-based VIGS system that could successfully induce yellow leaf phenotypes by silencing *LcCLA1* and *LcPDS*, respectively. According to the influencing factors such as *Agrobacterium* strain GV3101, tip needle injection method and inoculated plant growth temperature of 26 °C, the relative expression level of *LcCLA1* and *LcPDS* can reach about 25–50%, respectively. The TRV-mediated VIGS system developed and used in this study provides an alternative tool for functional gene studies in *L. chinensis*. This study also could provide a reference for the development of a rapid, transient and stable transformation system for *Lycoris*. In addition, we have improved the injection method for blades with waxy surfaces, which greatly improves the injection efficiency and ease of operation. This can provide new ideas for other plants that are not easily penetrated. These results help to lay the foundation for gene expression analysis in *Lycoris* and better studying of the molecular mechanism of related traits.

## Figures and Tables

**Table 1:** List of primers used in this study.

Primer Name	Primer Sequence
*EXP1*-RT-F	TTGATGTTGACAAGGTAAGGTGC
*EXP1*-RT-R	AGGCAGGAAATCTCCAAAGC
*LcCLA1*-F	TTGACGGGCAGGAGGGAC
*LcCLA1*-R	GCGGAGGAAGCAGTTTAGGC
*LcCLA1*-XbaI-F	CTAGTCTAGATTGACGGGCAGGAGGGAC
*LcCLA1*-KpnI-R	CGGGGTACCGCGGAGGAAGCAGTTTAGGC
*LcCLA1*-RT-F	GTTGCTCATTTCTTGGCACTCA
*LcCLA1*-RT-R	CAGCACCAACGGTCTCCACT
*LcPDS*-F	GTTAGGTCAGTTTCTGCTGTTTGTC
*LcPDS*-R	GTTGTTCCTCAAGATAGCCCATA
*LcPDS*-XbaI-F	CTAGTCTAGAGTTAGGTCAGTTTCTGCTGTTTGTC
*LcPDS*-KpnI-R	CGGGGTACCGTTGTTCCTCAAGATAGCCCATA
*LcPDS*-RT-F	AAAACCGTACCCGACTGTGAG
*LcPDS*-RT-R	CGGCTGTAGACACTTTCTTGCT

## Data Availability

The data are available from the corresponding author upon request.
